# Auxotrophy-based High Throughput Screening assay for the identification of *Bacillus subtilis* stringent response inhibitors

**DOI:** 10.1038/srep35824

**Published:** 2016-10-24

**Authors:** Liis Andresen, Vallo Varik, Yuzuru Tozawa, Steffi Jimmy, Stina Lindberg, Tanel Tenson, Vasili Hauryliuk

**Affiliations:** 1 Department of Molecular Biology, Umeå University, Building 6K, 6L University Hospital Area, SE-901 87 Umeå, Sweden; 2 Laboratory for Molecular Infection Medicine Sweden (MIMS), Umeå University, Building 6K and 6L, University Hospital Area, SE-901 87 Umeå, Sweden; 3 University of Tartu, Institute of Technology, Nooruse 1, 50411 Tartu, Estonia; 4 Graduate School of Science and Engineering, Saitama University, 255 Shimo-Okubo, Sakura-ku, Saitama, Saitama 338-8570, Japan; 5 Laboratories for Chemical Biology Umeå (LCBU), Department of Chemistry, Umeå University, SE-901 87 Sweden

## Abstract

The stringent response is a central adaptation mechanism that allows bacteria to adjust their growth and metabolism according to environmental conditions. The functionality of the stringent response is crucial for bacterial virulence, survival during host invasion as well as antibiotic resistance and tolerance. Therefore, specific inhibitors of the stringent response hold great promise as molecular tools for disarming and pacifying bacterial pathogens. By taking advantage of the valine amino acid auxotrophy of the *Bacillus subtilis* stringent response-deficient strain, we have set up a High Throughput Screening assay for the identification of stringent response inhibitors. By screening 17,500 compounds, we have identified a novel class of antibacterials based on the 4-(6-(phenoxy)alkyl)-3,5-dimethyl-1H-pyrazole core. Detailed characterization of the hit compounds as well as two previously identified promising stringent response inhibitors – a ppGpp-mimic nucleotide Relacin and cationic peptide 1018 – showed that neither of the compounds is sufficiently specific, thus motivating future application of our screening assay to larger and more diverse molecular libraries.

The stringent response is a central adaptation mechanism that adjusts bacterial growth and metabolism to environmental conditions. In response to various stress stimuli, RelA/SpoT Homologue (RSH) proteins modulate the intracellular concentration of the nucleotide alarmone guanosine (penta)tetraphosphate or (p)ppGpp^1^. An increased level of (p)ppGpp effectuates the adaptation to stress conditions via a global rewiring of the cellular metabolism and transcriptional program, e.g. by upregulating the production of amino acid biosynthesis enzymes upon amino acid starvation^2^.

In the most commonly used bacterial model organism – γ-proteobacterium *Escherichia coli* – the stringent response is orchestrated by two multi-domain ‘long RSH’ enzymes: RelA^3^ and SpoT^4^. Their activity is regulated by different sets of stress signals. RelA has strong ribosome-dependent (p)ppGpp synthetic activity that is triggered upon amino acid starvation via RelA directly sensing the deacylated tRNA in the ribosomal A-site^5^,^6^,^7^,^8^,^9^. As we have shown using an *in vitro* biochemical system another activator of RelA is its product ppGpp^10^, though the physiological significance of this effect is not yet clear. The other *E. coli* RSH, SpoT, possesses both (p)ppGpp synthetic and hydrolytic activities^11^,^12^. The weak synthetic activity of SpoT is induced by a variety of signals including fatty acid^13^, iron^14^ and carbon-source^11^ starvation. Constitutive (p)ppGpp hydrolysis by SpoT is crucial for counteracting the toxic effects of (p)ppGpp overproduction, and therefore disruption of the *spoT* gene in the presence of an intact copy of the *relA* gene renders *E. coli* non-viable^11^.

Phylogenetic analysis of the RSH protein family has shown that RelA and SpoT have a very limited evolutionary distribution^1^. In the majority of bacterial species, including the well-studied model organism *Bacillus subtilis*, ‘long’ RSHs are represented by the protein Rel, the progenitor of RelA and SpoT^1^. Like SpoT, Rel has both synthetic and hydrolytic activities, and like RelA, its synthetic activity is stimulated by starved ribosomes harbouring a deacylated tRNA in the A-site^15^,^16^. In addition to the above-mentioned ‘long’ multi-domain RSHs – RelA, Rel and SpoT – there are more than twenty subfamilies of ‘short’ single-domain RSHs that possess only the synthetic (Small Alarmone Synthetase, SAS) or hydrolytic (Small Alarmone Hydrolase, SAH) domain^1^. *B. subtilis* possesses two SAS proteins: SAS1 (synonyms: YjbM and RelQ) and SAS2 (synonyms: YwaC and RelP)^17^,^18^,^19^. While under normal growth conditions SAS enzymes contribute to basal (p)ppGpp levels^20^, cell wall stress stimuli such as treatment with cell wall-active antibiotics or alkaline shock induce expression of SAS via transcriptional up-regulation, and the resultant increase in (p)ppGpp levels orchestrates the response to stress^17^,^21^.

The functionality of the (p)ppGpp-mediated regulatory system is crucial for bacterial virulence^22^, survival during host invasion^21^ and antibiotic tolerance^23^. The alarmone (p)ppGpp was recently proposed to be the primary driver behind the formation of antibiotic-tolerant phenotypic variants in clonal bacterial populations, known as persister cells^24^. All this, in combination with the absence of a cytoplasmic RSH-mediated stringent response system in eukaryotes^1^,^25^, makes the enzymes involved in (p)ppGpp metabolism promising new targets for drug discovery, as inhibitors of the stringent response would act as anti-virulence agents. Disarming the pathogens, and targeting bacterial virulence – rather than killing bacteria – is believed to be a promising strategy due to lower selection pressure leading to slower emergence of resistance^26^.

The first steps towards the development of a specific and potent inhibitor of the stringent response have already been taken with the development of a nucleotide-based RSH inhibitor, Relacin^27^, and the anti-biofilm peptide 1018 that was suggested to inhibit the stringent response by binding (p)ppGpp and promoting its degradation^28^. However, Relacin is rather inefficient – it requires sub-mM concentrations^27^,^29^ – and 1018 has a strong bacteriotoxic effect; the concentration range in which it transitions from merely dispersing biofilms to killing bacteria is approximately 10-fold^28^.

Therefore, there is a need for more potent and selective stringent response inhibitors, motivating the current High Throughput Screening (HTS) project. Our HTS strategy is based on the following considerations. First, we opted for a whole-cell assay instead of an enzyme-based one, since inefficient cellular uptake is one of the main challenges in the discovery of novel antibacterials^30^,^31^. Second, we chose a phenotype-based screening approach – a strategy designed for the identification of compounds that target a specific pathway rather than antibacterials in general^32^.

## Results

### Screening strategy for the identification of *B. subtilis* Rel inhibitors relying on amino acid auxotrophy

We chose the Gram-positive bacterium *B. subtilis* to be used in the screening process because the chances of identifying biologically active compounds in Gram-positive bacteria are considerably higher than in Gram-negative bacteria^30^. To improve the selectivity of the HTS for the inhibition of long ribosomal RSH Rel – the primary driver of acute stringent response – we used a *B. subtilis* strain lacking functional SAS RelQ and RelP (Δ*SAS* strain)^17^. Moreover, SAS enzymes can be refractory to inhibitors of Rel, e. g. *Enterococcus faecalis* RelQ is insensitive to ppGpp analogue Relacin^29^, thus potentially masking the effect of Rel inhibition.

**Figure 1:**
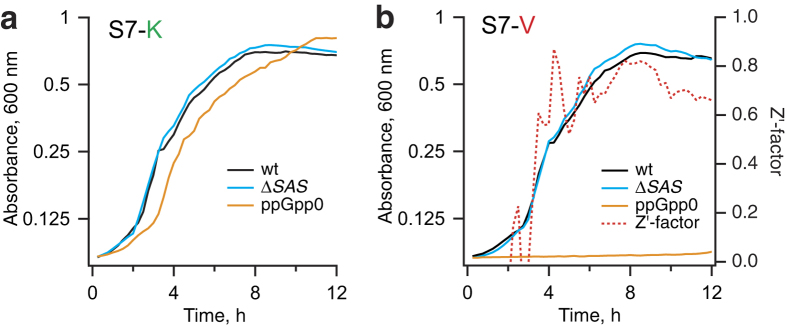
Valine autotrophy of ppGpp^0^ *B. subtilis* can be exploited for the detection of Rel inhibition. *B. subtilis* strain 168 (wt), its isogenic strain lacking Small Alarmone Synthetases (SAS) YjbM and YwaC (Δ*SAS*; RIK1002^17^) and a ppGpp-deficient strain lacking both SAS and long RSH Rel (ppGpp^0^; RIK1003^17^) were grown in S7 minimal medium supplemented with 1% glucose and a set of 19 amino acids lacking either L-lysine (**a**; S7-K) or L-valine (**b**; S7-V). (**a**) In S7 medium lacking lysine (S7-K) the ppGpp^0^ strain grows in a similar way to the isogenic wild type and Δ*SAS*. (**b**) Due to valine autotrophy, ppGpp^0^ *B. subtilis* is unable to grow in S7-V while the Δ*SAS* strain grows as well as the wild type. Traces show arithmetic mean OD_600_ values of four technical replicates; means and standard deviations of OD_600_ readings were used to calculate the Z’-factor^35^.

Our screening assay relies on the amino acid auxotrophy phenotype characteristic of (p)ppGpp-deficient (ppGpp^0^) strains^11^,^33^. Specifically, we exploit the auxotrophy of ppGpp^0^ *B. subtilis* for branched-chain amino acids – valine, leucine and isoleucine^33^ – by using a combination of two drop-out variants of S7 growth medium^34^: S7 lacking valine (S7-V) and S7 lacking lysine (S7-K). Wild type, Δ*SAS* and ppGpp^0^ *B. subtilis* (Δ*SAS*Δ*rel*) strains grew efficiently on S7-K (Fig. 1a). However, omission of valine completely inhibits the growth of the ppGpp^0^ strain without significantly affecting the growth of the isogenic wild type and the Δ*SAS* strains (Fig. 1b). The growth assay differentiates between Δ*SAS* and ppGpp^0^ strains with a Z’-factor^35^ exceeding 0.5 when the OD_600_ of the Δ*SAS* strain reaches 0.25 and above, and is readily adaptable to the 384-well screening format (Supplementary Fig. S1).

### Two-stage HTS for Rel inhibitors

To identify potential Rel inhibitors, we screened for a conditional growth inhibition of the Δ*SAS* strain in S7-V medium, but not in S7-K medium. The conditionality of the growth inhibition is used to sieve out generally cytotoxic compounds. S7-V-specific growth inhibition can, potentially, occur for several reasons. It may be due to the inhibition of the stringent response – the desired outcome – or be due for an unrelated reason, e.g. the compound is directly targeting an enzyme in valine biosynthesis leading to the absence of valine or is affecting either GTP biosynthesis or GTP sensing thus phenocopying ppGpp^0^ 33,36,37,38,39. Even the desired outcome – inhibition of (p)ppGpp accumulation – could, potentially, be brought about not via inhibition of Rel’s (p)ppGpp synthesis activity (the desired hit) but via activation of its hydrolytic activity; cross-talk between the two active sites of the enzyme^40^ can further complicate matters. Therefore, it is impossible to estimate *a priori* the proportion of false positive hits generated by the screen and it is crucial to directly test the efficiency of identified compounds against the desired molecular target, i. e. the (p)ppGpp synthesis activity of *B. subtilis* Rel.

**Figure 2:**
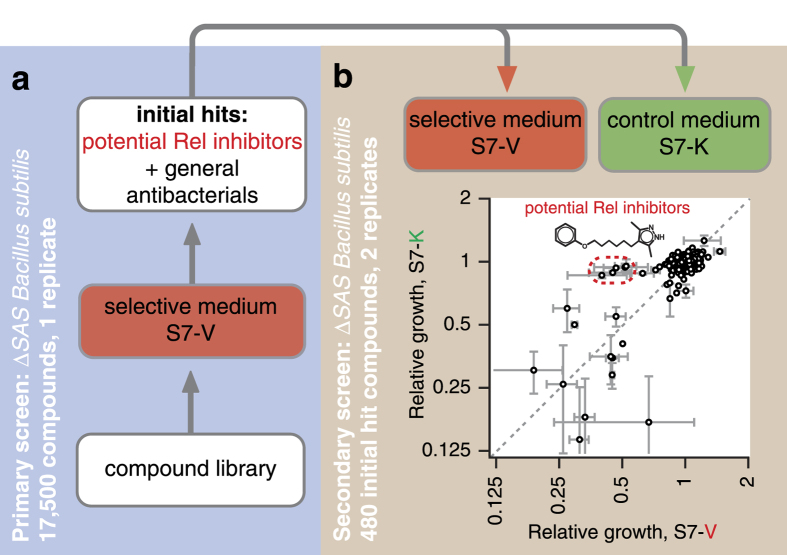
Two-stage HTS for Rel inhibitors. *B. subtilis* Δ*SAS* was grown on 384-well plates in the presence of 10 μM of each test compound. OD_600_ values were scored after 9 hours of growth at 37 °C. (**a**) The primary screen was performed in S7-V medium using 17,500 compounds selected from a set of small molecule libraries (ChemBridge). Compounds that inhibited *B. subtilis* growth in S7-V were scored using MScreen^42^ and a custom R script (see **Methods**), the lists of initial hit compounds identified by the two approaches were pooled and analysed by the secondary screen. (**b**) In the secondary screen 480 initial hits from the primary screen were screened in both S7-V (selective medium) and S7-K (control medium for identification of general antibacterials) in two replicates. Results fall into the following three categories: selective inhibitors causing valine auxotrophy, e.g. via inhibition of Rel; general antibacterials; and random fluctuations causing growth retardation. Five compounds, all sharing the common 4-(6-(phenoxy)alkyl)-3,5-dimethyl-1H-pyrazole moiety (see Table 1), selectively inhibited growth in S7-V, but not in S7-K (encircled with a red dashed line).

We based our HTS assay on the protocol by Zlitni and colleagues^41^. Frozen stock of the *B. subtilis* Δ*SAS* strain was used as a starting material by diluting the cell suspension ten times in the respective medium. For the primary screen we used the LCBU Screening Set of 17,500 synthetic drug-like organic compounds in S7-V medium at a final concentration of 10 μM in the presence of 0.9% DMSO (Fig. 2a). The growth of the Δ*SAS* strain was scored after 9 hours at 37 °C using the absorbance at 600 nm as a readout. Compounds inhibiting bacterial growth were identified using two complementary approaches: MScreen^42^ and our custom pipeline (see Methods for details). MScreen calculates mean value for all sample wells plate-by-plate and rates compounds according to their relative Z-score^35^. Our pipeline identifies and ranks local outliers using median absolute deviation (MAD) of absorbance values within a sliding window as a measure of local variability (see Supplementary Fig. S2 for the graphical output of the in-house program). The 300 top scoring candidates identified by the two approaches were pooled together yielding a final list of 480 hits candidate compounds after the removal of duplicates (see Supplementary Fig. S3 for a comparison of the two approaches).

In the secondary screening we tested the 480 initial hits for growth retardation of Δ*SAS B. subtilis* in both S7-V (the selective medium) and S7-K (the control medium for identification of general antibacterials) (Fig. 2b). Since the readout – growth retardation – is prone to the generation of spurious hits, we performed two replicates of the secondary screening. The two replicates showed satisfactory reproducibility with Pearson’s product-moment correlation coefficient above 0.8 (Supplementary Fig. S4).

Twelve compounds displayed equally pronounced inhibitory effects in both S7-V and S7-K, suggesting that these are general antibacterials and are not of interest as potential stringent response inhibitors (Supplementary Table 1). However, five compounds have a moderate inhibitory effect on *B. subtilis* growth in S7-V (growth retardation of approximately 50%) while they have virtually no effect on growth in S7-K (Fig. 2b, encircled in red dashed line). The identified compounds are: 4-[6-(4-chlorophenoxy)hexyl]-3,5-dimethyl-1H-pyrazole (**1,** C302), 4-[6-(2,5-dimethylphenoxy)hexyl]-3,5-dimethyl-1H-pyrazole (**2,** C318), 3,5-dimethyl-4-[6-(2-methylphenoxy)hexyl]-1H-pyrazole (**3,** C303), 4-[6-(3,5-dimethylphenoxy)hexyl]-3,5-dimethyl-1H-pyrazole (**4,** C285), and 4-[4-(2-tert-butylphenoxy)butyl]-3,5-dimethyl-1H-pyrazole (**5,** C385) (Table 1). Importantly, all compounds share the same core, 4-(6-alkyl)-3,5-dimethyl-1H-pyrazole, and do not possess any of the characteristics of Pan Assay Interference compounds (PAINS) – a group of structurally diverse compounds displaying nonspecific activity against a wide range of unrelated target proteins^43^,^44^.

### Derivatives 4-(6-(phenoxy)alkyl)-3,5-dimethyl-1H-pyrazole are general antibacterials

To investigate the specificity of the five identified hit compounds against the stringent response, we characterized their efficiency in dose-response assays (Supplementary Fig. S5). Disappointingly, as the concentrations increase all of the compounds efficiently inhibit *B. subtilis* growth not just in the selective S7-V medium, but also in the control S7-K medium. The half maximal inhibitory concentrations (IC_50_) range from 4.5 to 14.5 μM, and the concentration used for the HTS (10 μM) is located on the slope of the dose-response curve where the effect is extremely sensitive to assay conditions such as composition of the growth medium.

**Figure 3:**
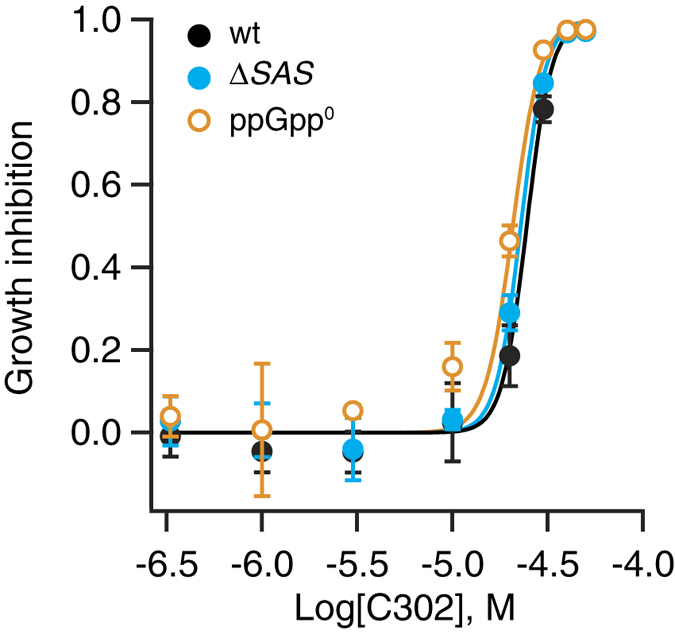
Growth inhibition efficiency of compound C302 against *B. subtilis* ppGpp-synthetase mutants. A representative of the HTS hit compounds, compound C302 (4-[6-(4-chlorophenoxy)hexyl]-3,5-dimethyl-1H-pyrazole), was serially diluted in DMSO and titrated into liquid cultures of *B. subtilis* wild-type (wt) strain, mutant strain lacking YjbM and YwaC (Δ*SAS*), and ppGpp-deficient (ppGpp^0^) strains. The growth conditions are identical to those used for HTS with the exception of the S7 medium being supplemented with the full set of 20 amino acids and 1.3% DMSO. Half-maximal inhibitory concentrations are 24.5 μM (CI95% 23.3–25.9) for wt, 22.8 μM (CI95% 21.8–23.9) for Δ*SAS* and 20.7 μM (CI95% 19.3–22.3) for ppGpp^0^. The experiments were performed in three replicates and error bars (too small to be seen for some of the points) indicate standard error of the mean.

To confirm that Rel is not the primary target of the hit compounds, we have tested a representative hit compound – C302 – against wild type, Δ*SAS* and ppGpp^0^ *B. subtilis* strains in S7 medium supplemented with the full set of 20 amino acids in order to mitigate the potential metabolic differences between the strains (Fig. 3). Growth of all strains is inhibited by C302 with an IC_50_ around 20–25 μM, though ppGpp^0^ *B. subtilis* is marginally more sensitive than the other two strains. Finally, we directly tested the effect of C302 on ppGpp synthesis by using purified *B. subtilis* Rel activated by ‘starved’ ribosomal complexes carrying deacylated tRNA in the A-site; we observed no inhibition (Supplementary Fig. S6). Taken together, our data suggest that rather than acting as specific Rel inhibitors, the derivatives of 4-(6-(phenoxy)alkyl)-3,5-dimethyl-1H-pyrazole act as general antibacterials.

### Relacin and 1018 do not pass the valine auxotrophy test for specific inhibitors of the stringent response in *B. subtilis*

**Figure 4:**
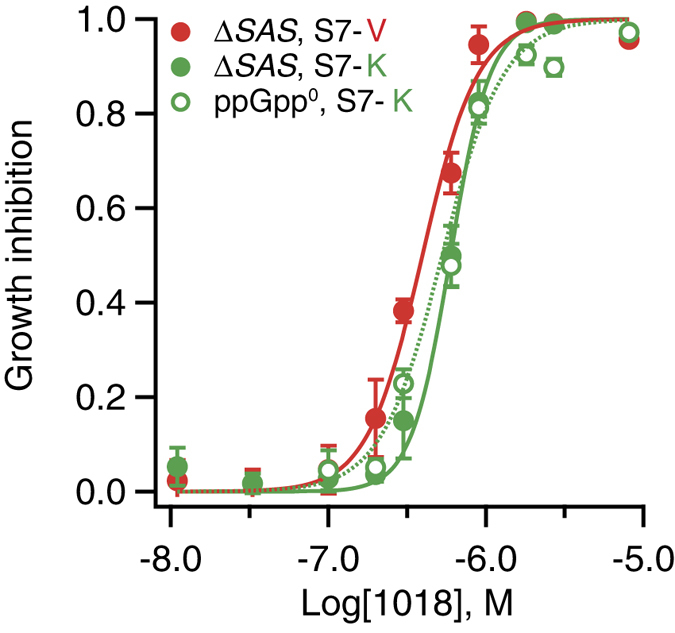
Peptide 1018 does not pass the selection criteria for a selective Rel inhibitor of the current auxotrophy-based HTS. Dose-response curves of the *B. subtilis* Δ*SAS* strain in S7-K and the *B. subtilis* ppGpp^0^ strain in S7-K in the presence of increasing concentrations of peptide 1018. Peptide 1018 has the same growth inhibitory efficiency against Δ*SAS* and ppGpp^0^ strains in S7-K with an IC_50_ of 0.6 μM (CI95% 0.56–0.63) and 0.53 μM (CI95% 0.48–0.59), respectively. In S7-V medium the ΔSAS strain is slightly more sensitive to the peptide treatment with an IC_50_ of 0.4 μM (CI95% 0.36–0.44)). The experiments were performed in three replicates and error bars (too small to be seen for some of the points) indicate standard error of the mean.

We have benchmarked our assay against the two previously reported inhibitors of the stringent response: the anti-biofilm peptide 1018^28^ and the ppGpp-based nucleotide Relacin^27^. When tested against the Δ*SAS* strain, the addition of up to 2 mM of Relacin does not inhibit the bacterial growth in either S7-V or S7-K medium (Supplementary Fig. S7). Similarly, the compound has little effect on the growth of the ppGpp^0^ strain (Supplementary Fig. S7). Peptide 1018 efficiently inhibits *B. subtilis* growth in the S7-V medium with half of the maximal inhibitory concentration IC_50_ = 0.4 μM (CI 95%: 0.36–0.44), whereas in S7-K medium the bacterium is slightly less sensitive to the inhibitor (IC_50_ = 0.6 μM; CI 95%: 0.56–0.63) – a similar effect has lead to the identification of the compounds in the current HTS (Fig. 4). Moreover, 1018 has essentially the same growth inhibition efficiency on the ppGpp^0^ strain in S7-K (IC_50_ = 0.53 μM, CI95%: 0.48–0.59), underscoring the ppGpp-independent nature of the antibacterial effect.

## Discussion

Despite relying on a robust, virtually “all-or-nothing” auxotrophic phenotype of the ppGpp^0^ *B. subtilis* strain, our HTS project did not yield a selective stringent response inhibitor. A class of compounds – derivatives of 4-(6-(phenoxy)alkyl)-3,5-dimethyl-1H-pyrazole – did display preferential activity against *B. subtilis* growing in the medium lacking valine, as compared to the medium lacking lysine in a single-concentration HTS assay. However, subsequent dose-response assays revealed a dominant general antibacterial effect. Further derivatization and structure activity relationship analysis is necessary to assess the potential of 4-(6-(phenoxy)alkyl)-3,5-dimethyl-1H-pyrazoles as antibacterials. We have used our screening assay to test two promising stringent response inhibitors – a ppGpp-mimic nucleotide, Relacin,^27^ and an antibiofilm peptide, 1018^28^ – and neither of the two pass the stringent selection criteria used in the current study. The efficiency of both compounds against *B. subtilis* is virtually the same for ppGpp-deficient and wild type strains (Fig. 4, Supplementary Fig. S7). The quest for the discovery of specific and potent stringent response inhibitors continues.

Since our HTS assay is calibrated against a complete genetic disruption of the ppGpp synthesis in the cell, the potential hit compounds are expected to be both efficient and specific. Unfortunately, such molecules were absent in the screening library employed in the current study. This is a common problem: conventional chemical libraries used for HTS projects are ill-suited for antibiotic discovery since, as judged by molecular weight and polarity, natural antibiotics poorly fit the molecular profile of the drug-like molecules used to populate the chemical libraries^30^,^45^. Development of targeted HTS libraries with validated bacterial permeability could considerably improve the hit rate. However, we are not aware of the existence of such libraries. Therefore, application of the HTS assay described in the current report to alternative sources of compounds such as natural products^46^ is a possible next step.

## Methods

### *Bacillus subtilis* strains and growth conditions

Construction of the *B. subtilis* 168-based strain RIK1002 lacking Small Alarmone Synthetases (SAS) YjbM and YwaC (ΔSAS; *trpC2* Δ*yjbM ywaC*::*spc*) and the ppGpp-deficient strain RIK1003 additionally lacking Rel (ppGpp^0^; *trpC2* Δ*yjbM ywaC*::*spc relA*::*erm*) is described in Nanamiya *et al*.^17^. Liquid cultures were grown at 37 °C in S7 minimal medium supplemented with 1% glucose and amino acids as per Nicholson and Seltow^47^. The medium was prepared as described in Nanamiya *et al*.^17^ except L-glutamate was substituted with a full set of 20 naturally-occurring amino acids as per Cutting and Horn^34^; L-valine (S7-V) or L-lysine (S7-K) were omitted to generate selective media for screening. Cultures were grown with aeration for the preparation of cell stocks and without aeration during HTS. To prepare the bacterial stock for inoculation for HTS *B. subtilis* strains RIK1002 and RIK1003 were grown from a single fresh colony to OD_600_ 1.7 in S7 medium supplemented with the full set of 20 amino acids (S7), diluted to OD_600_ 0.25 in S7-V or S7-K, supplemented with 8% DMSO, aliquoted, snap frozen in liquid nitrogen and stored at −70 °C prior to the screen for not longer than 6 weeks.

### Primary and secondary HTS

The HTS procedure was based on the protocol by Zlitni and colleagues^41^. The primary screen used a LCBU Screening Set of 17,500 compounds selected from a set of small molecule libraries (ChemBridge) dispensed using Echo®-(Labcyte) directly into 384-well plates yielding 56 plates with 320 compounds per plate with the first two and last two columns reserved for controls, 80 nl of 10 mM compound in 100% DMSO was added per well, and 80 nl of 100% DMSO was used in control wells. Frozen stocks of RIK1002 were thawed on ice, diluted 10 times with S7-V medium and 80 μl was dispensed per well on library plates (final concentration of compounds: 10 μM and DMSO of 0.9%). The ppGpp^0^ strain RIK1003 served as a positive control (10 wells, 80 μl per well) and Δ*SAS* strain RIK1002 as a negative control (16 wells, 80 μl per well). The first and the last column contained pure medium as a contamination control. Plate lids were sealed with parafilm, stacked by four and incubated at 37 °C for 9 hours. Evaporation from the plates during the incubation at 37 °C was countered in two ways. First, plates were stacked by four and each stack was topped with an extra plate filled with water. Second, a water bath was placed in the incubator. Growth was measured at OD_600_. For the secondary screen using a combination of S7–V and S7–K media, 480 compounds identified as possible hits (see below, *HTS data analysis*) during the primary screen were tested in two replicates. As an extra precaution against the edge effects the two outer-most columns of each plate were left empty.

### HTS data analysis

Statistical analysis of HTS results followed the guidelines of Malo and colleagues^48^. Primary screen data were analysed with a custom script in the R programming language^49^ and with MScreen^42^. The R script compares the read out of the individual well with the median of a window of wells, calculated in median absolute deviations (MAD) (the code is provided in the Supplementary Information). MScreen compares the read out of the individual well with the average of sample wells on the whole plate, calculated in standard deviations (SD)^42^. The 300 top candidates identified by the two programs were pooled together yielding, after removal of duplicates, a final list of 480 hits used for the secondary screen. The performance of the two approaches is compared in Supplementary Fig. S3. Identity of the hit compounds was confirmed by liquid chromatography-mass spectrometry (LC-MS).

### Dose-response analysis

For dose-response analysis, the HTS assay was performed in a 96 well microtiter plate format using 160 μl of ΔSAS *B. subtilis* cultures supplemented with increasing concentrations of test compound in the presence of 1.3% DMSO. Bacterial growth was measured at OD_600_ after 9 hours of incubation at 37 °C. Growth inhibition was calculated as 1 – (A_S_ – A_M_)/(A_UT_-A_M_) where A stands for OD_600_ absorbance of the well and indexes S, M, UT indicate sample, medium and untreated control, respectively. Dose-response curves and IC_50_ values together with 95% confidence intervals were calculated in Prism 6 (GraphPad) using the variable slope Hill equation (Y = Bottom + (Top-Bottom)/(1 + 10^((LogIC_50_-X)*HillSlope)) where Y is the modelled response; Bottom is the lowest experimental growth inhibition value; Top is the highest experimental growth inhibition value; IC_50_ is the half-maximal inhibitory concentration; and X is the compound concentration. Relacin was synthesized as per Gaca *et al*. (2015)^29^. Peptide 1018 (VRLIVAVRIWRR-NH_2_) was ordered in lyophilized form from Storkbio Ltd (>95% pure).

### *B. subtilis* Rel enzymatic assay

The ppGpp synthesis assay was performed as per Shyp *et al*. (2012)^10^ with *B. subtilis* Rel activated by *B. subtilis* ribosomes with dealylated tRNA^Phe^ in the presence of 100 μM pppGpp and using 1 mM ATP and 300 μM ^3^H-GTP as substrates. For more details, see Supplementary Information.

## Additional Information

**How to cite this article**: Andresen, L. *et al*. Auxotrophy-based High Throughput Screening assay for the identification of *Bacillus subtilis* stringent response inhibitors. *Sci. Rep.* **6**, 35824; doi: 10.1038/srep35824 (2016).

## Supplementary Material

Supplementary Information

## Figures and Tables

**Table 1:**
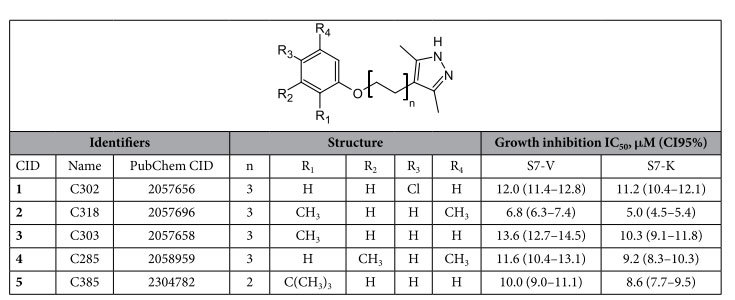
Structure and growth inhibition efficiency of the hit compounds.

Our two-stage screen resulted in five hits with a common structural core, 4-(6-(phenoxy)alkyl)-3,5-dimethyl-1H-pyrazole. Based on dose-response analysis, none of the compounds inhibit growth specifically in S7 medium lacking valine (S7-V) when compared to the medium lacking lysine (S7-K). Dose-response analyses were performed in three technical replicates. IC_50_: half-maximal inhibitory concentration; CI95%: 95% confidence interval.
