# Supplementary material for: Auxotrophy-based High Throughput Screening assay for the identification of Bacillus subtilis stringent response inhibitors
Source: Sci Rep. 2016 Oct 24;6:35824. doi: 10.1038/srep35824 (PMC5075769; doi:10.1038/srep35824)
Supplement: Supplementary Information [file srep35824-s1.pdf]

Supplementary Information

Auxotrophy-based High Throughput Screening assay for the identification of *Bacillus subtilis* stringent response inhibitors

Liis Andresen[1,2,⌘,*], Vallo Varik[1,2,3,⌘], Yuzuru Tozawa[4], Steffi Jimmy[1,2], Stina Lindberg[5], Tanel Tenson[3] & Vasili Hauryliuk[1,2,3,*]

[1]Department of Molecular Biology, Umeå University, Building 6K, 6L University Hospital Area, SE-901 87 Umeå, Sweden
[2]Laboratory for Molecular Infection Medicine Sweden (MIMS), Umeå University, Building 6K and 6L, University Hospital Area, SE-901 87 Umeå, Sweden
[3]University of Tartu, Institute of Technology, Nooruse 1, 50411 Tartu, Estonia
[4]Graduate School of Science and Engineering, Saitama University, 255 Shimo-Okubo, Sakura-ku, Saitama, Saitama 338-8570, Japan.
[5]Laboratories for Chemical Biology Umeå (LCBU), Department of Chemistry, Umeå University, SE-901 87 Sweden
[⌘]denotes equal contribution
[*]denotes corresponding authors

Contact details of corresponding authors:
Liis Andresen: liis.andresen@umu.se, +46702016884
Vasili Hauryliuk: vasili.hauryliuk@umu.se, +46907850807

Supplementary Methods

Preparation of B. subtilis *Rel*

B. subtilis rel gene coding for (p)ppGpp synthetase was cloned into pET24d plasmid. The protein was overexpressed in *E. coli* BL21(DE3) from pET284d-*rel* plasmid as follows. Single colony was inoculated into 3 ml of LB medium and grown aerobically at 37°C until OD_{600}=0.5 in the presence of 50 µg/ml kanamycin (Km). The culture was transferred into 400 ml of fresh pre-warmed 2xYT medium (with 50 µg/ml Km) and grown aerobically at 37°C to OD_{600}=0.5. Rel overexpression was induced by addition of IPTG with a final concentration of 1 mM to the culture. The temperature was decreased to 30°C and bacteria were grown for another 1 hour. The bacterial cell mass was collected by centrifugation, resuspended in lysis buffer (50 mM NaH_2PO_4 pH 7.5, 350 mM NaCl, 2 mM $MgCl_2$, 10 mM imidazole, 1 mM 2-mercaptoethanol (βME), 100 µM PMSF and 1 U/ml DNase I) and lysed with Stansted SPCH-10 homogenizer. The lysate was centrifuged and the supernatant was used for Zn-NTA Sepharose affinity chromatography. The elution buffer consisted of 50 mM NaH_2PO_4 pH 7.5, 1 M NaCl, 2 mM $MgCl_2$, 10 mM imidazole, and 1 mM βME. The peak corresponding to Rel protein was collected and loaded on gel filtration column (HiLoad 16/600, Superdex 200 pg). Purified protein was concentrated using Amicon Ultra-15 (Millipore) centrifugal filter devices with a 50 kDa cut-off. The final preparations were aliquoted, snap-frozen with liquid nitrogen and stored at –80°C in storage buffer (25 mM HEPES-KOH, pH 7.5, 0.7 M KCl, 1 mM βME, 5 mM $MgCl_2$ and 10% glycerol).

Enzymatic assays with B. subtilis *Rel*

ppGpp synthesis reactions were performed in 1x HEPES:Polymix buffer (25 mM HEPES, 15 mM Mg^{2+} and 1 mM βME) and contained 0.5 µM *B. subtilis* ribosomes, 100 µM ppGpp, 300 µM ^{3}H-labelled GDP, 0.1 µM Rel, 0.12 µg/ml polyU mRNA, 2 µM $tRNA^{Phe}$ and 0-50 µM compound C302. The reaction mixture was pre-incubated at 37°C for 2 min and started by addition of 1 mM ATP. At each time points 5 µl of reaction mixture was quenched with 70% formic acid and analyzed by TLC.

Thin layer chromatography (TLC) analysis of nucleotide mixtures

Rel ppGpp synthesis activity was followed by separating reaction mixtures on TLC analysis followed by scintillation counting according to Mechold and colleagues, with modifications[1]. At each time point 5 µl of reaction mixture was quenched by addition of 4 µl 70% formic acid supplemented with a cold nucleotide standard used for UV-shadowing (10 mM GDP and 10 mM GTP) and spotted on PEI-TLC plates (Macherey-Nagel). TLC was run in 0.5 M KH_2PO_4. The plates were dried and cut into sections guided by UV-shadowing and ^{3}H radioactivity was quantified by scintillation counting. Conversion of substrate to product was quantified as described in the Shyp et al. (2012)[2].

R code for detection of growth inhibition

```
                          ### Load required libraries ###
library(readxl); library(plyr); library(dplyr); library(zoo)

                          ### Read in and clean the data ###
temp <- list.files(pattern="*.xls")               # Point to the files
raw_data <- lapply(temp, read_excel)
raw_df <- ldply(raw_data, data.frame) # Turn into one data frame
df <- raw_df[ , c(3, 6)]                  # Take well ID and OD value
df <- cbind(Plate = rep(1:(length(df[,1])/384), each=384,), df)          # Plate index
df <- rename(df, OD = Abs600..0.1s...A.)          # Give a clean name to OD column

                              ### Split the data ###
# Set data filters for medium, dSAS, ppGpp0
pos.medium <- paste(LETTERS[1:16], rep(c("01", "24"), each=16), sep="")
pos.dSAS <- paste(LETTERS[1:16], rep("02", 16), sep="")
pos.ppGpp0 <- paste(LETTERS[1:16], rep("23", 16), sep="")
# Take compound wells
df_cmp <- filter(df, !Well %in% c(pos.medium, pos.dSAS, pos.ppGpp0))
df_cmp <- cbind(PlateWell=paste(df_cmp[,1],
        df_cmp[,2], sep="_"), OD=df_cmp[3])      # Merge, plate_well index

            ### Find local outliers by Median Absolute Deviation (MAD) threshold ###
# MAD function to identify local outliers
localt <- function(x) {m = median(x); m - threshold * median(abs(x - m))}
window <- 19                   # Set an arbitrary window, how many adjacent wells?
threshold <- 8                 # Set an arbitrary threshold, how many times MAD?
# Calculate threshold for each well
zt <- rollapply(df_cmp[,2], window, localt, align="center")
# Fill in start and end gaps, quick and dirty
zt <- c(rep(zt[1], window/2), zt, rep(zt[length(zt)], window/2))

                       ### Assign local outliers a MAD based score ###
# MAD function without threshold
localmad <- function(x) {m = median(x); m - median(abs(x - m))}
zmad <- rollapply(df_cmp[,2], window, localmad, align="center")
zmad <- c(rep(zmad[1], window/2), zmad, rep(zmad[length(zmad)], window/2))
zmed <- rollapply(df_cmp[,2], window, median, align="center")
zmed <- c(rep(zmed[1], window/2), zmed, rep(zmed[length(zmed)], window/2))
mad.score <- (zmed - df_cmp[,2])/(zmed - zmad)        # MAD score
df_cmp <- cbind(df_cmp, "Score"=mad.score)  # Add MAD score to data frame

                       #### Store and view outliers in a table ###
out <- filter(df_cmp, OD<zt); View(out, "Outliers")

                    ### Optional feature: plot MAD threshold outliers ###
plot(df_cmp$OD, type="l", lwd=1, col="red", ylab="OD")          # OD values
lines(seq_along(df_cmp$OD), zt[], col="gray")                   # Threshold
points(seq_along(df_cmp$OD)[df_cmp$OD<zt],
    filter(df_cmp, OD<zt)[[2]], pch=19)                         # Outliers
```

Description	Structure
① PubChem CID 460749<br>Rel. growth S7-V 0.026±0.053<br>Rel. growth S7-K 0.097±0.067	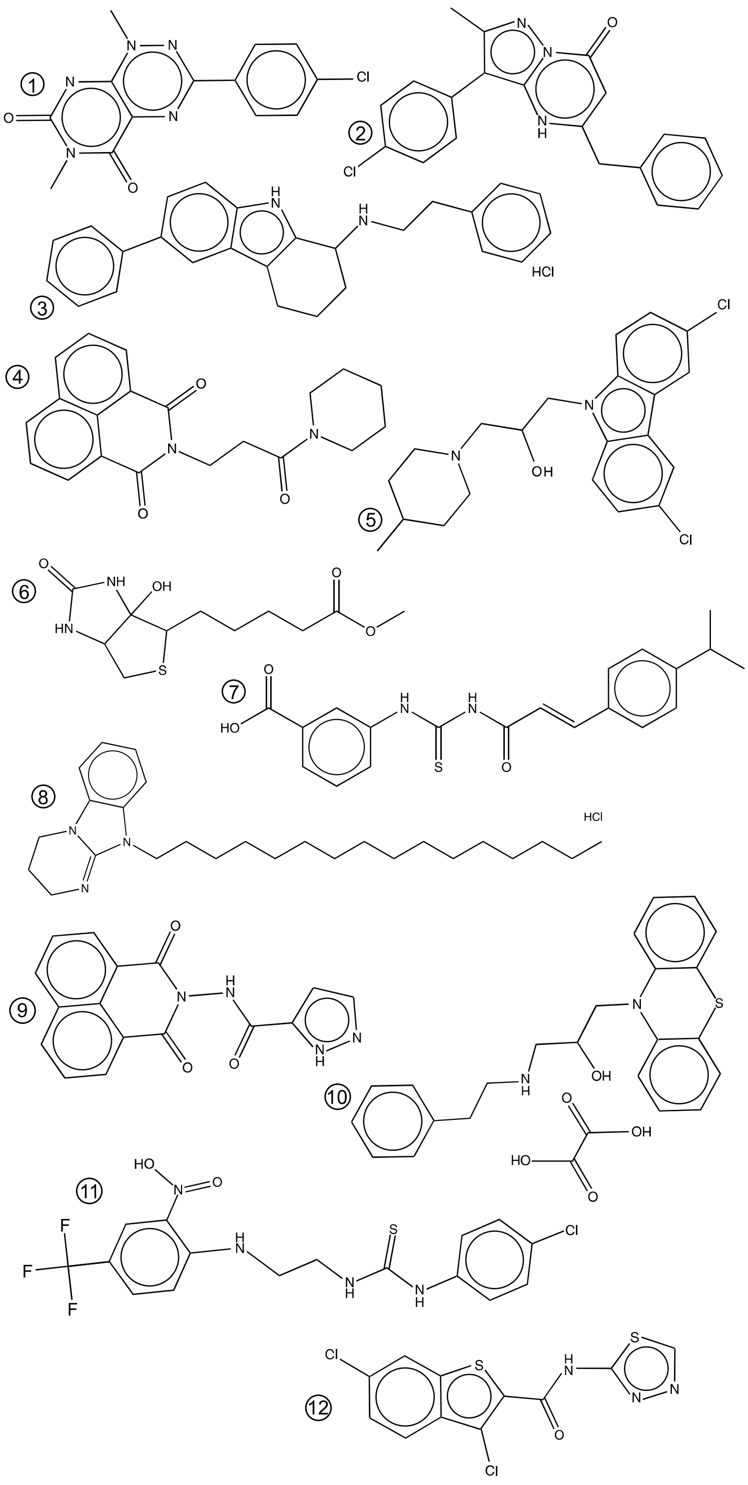
② PubChem CID 1280225<br>Rel. growth S7-V 0.313±0.033<br>Rel. growth S7-K 0.142±0.11	
③ PubChem CID 2838793<br>Rel. growth S7-V 0.189±0.07<br>Rel. growth S7-K 0.304±0.07	
④ PubChem CID 2838688<br>Rel. growth S7-V 0.332±0.037<br>Rel. growth S7-K 0.181±0.096	
⑤ PubChem CID 2861171<br>Rel. growth S7-V 0.447±0.01<br>Rel. growth S7-K 0.288±0.04	
⑥ PubChem CID 2849148<br>Rel. growth S7-V 0.449±0.032<br>Rel. growth S7-K 0.348±0.004	
⑦ PubChem CID 1327332<br>Rel. growth S7-V 0.261±0.043<br>Rel. growth S7-K 0.260±0.139	
⑧ PubChem CID 2851950<br>Rel. growth S7-V 0.441±0.09<br>Rel. growth S7-K 0.353±0.093	
⑨ PubChem CID 747531<br>Rel. growth S7-V 0.272±0.138<br>Rel. growth S7-K 0.599±0.039	
⑩ PubChem CID 2896977<br>Rel. growth S7-V 0.296±0.009<br>Rel. growth S7-K 0.501±0.007	
⑪ PubChem CID 2884570<br>Rel. growth S7-V 0.466±0.052<br>Rel. growth S7-K 0.549±0.058	
⑫ PubChem CID 1245053<br>Rel. growth S7-V 0.501±0.007<br>Rel. growth S7-K 0.406±0.004	

Supplementary Table 1 | Structures and growth effects of general antimicrobials. Our secondary screen identified 12 compounds that significantly inhibited *B. subtilis* growth in S7-V as well as S7-K media. Rel. growth indicates relative growth where 0 – no growth, 1 – growth of the untreated control. Values indicate mean values of relative growth of 2 replicates ± standard deviation.

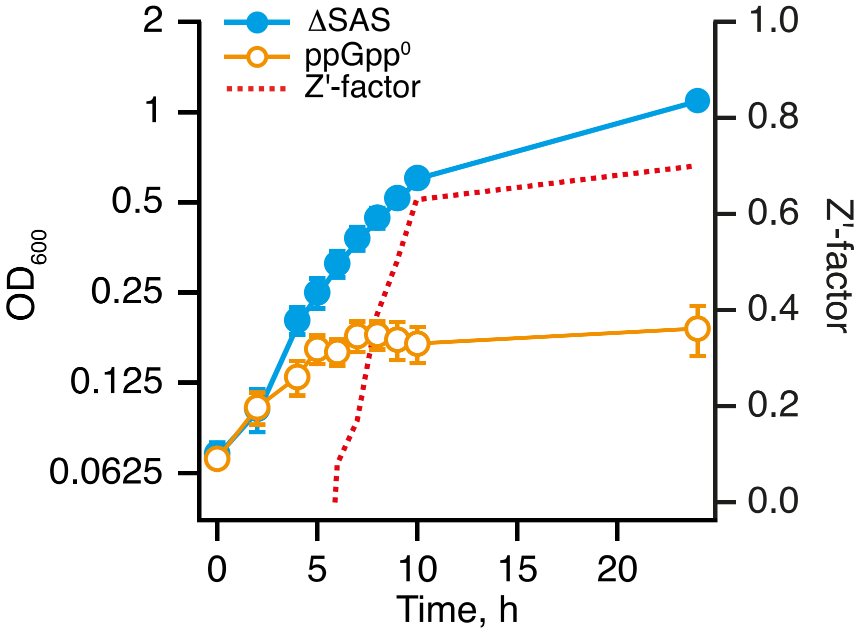

Supplementary Figure 1 | Auxotrophy-based HTS assay for Rel inhibitors in 384-well format. *B. subtilis* strain RIK1002[3] lacking SAS YjbM and YwaC (ΔSAS, blue filled circles) as well ppGpp-deficient strain RIK1003[3] lacking both SAS and long RSH Rel (ppGpp^0, red empty circles) were grown in S7 medium supplemented with 1% glucose and 19 amino acids mixture lacking L-valine (S7-V). Traces show an arithmetic mean OD_{600} values and standard deviations of 4 technical replicates. Z'-factor (red dashed line) was calculated according to Zhang and colleagues[4].

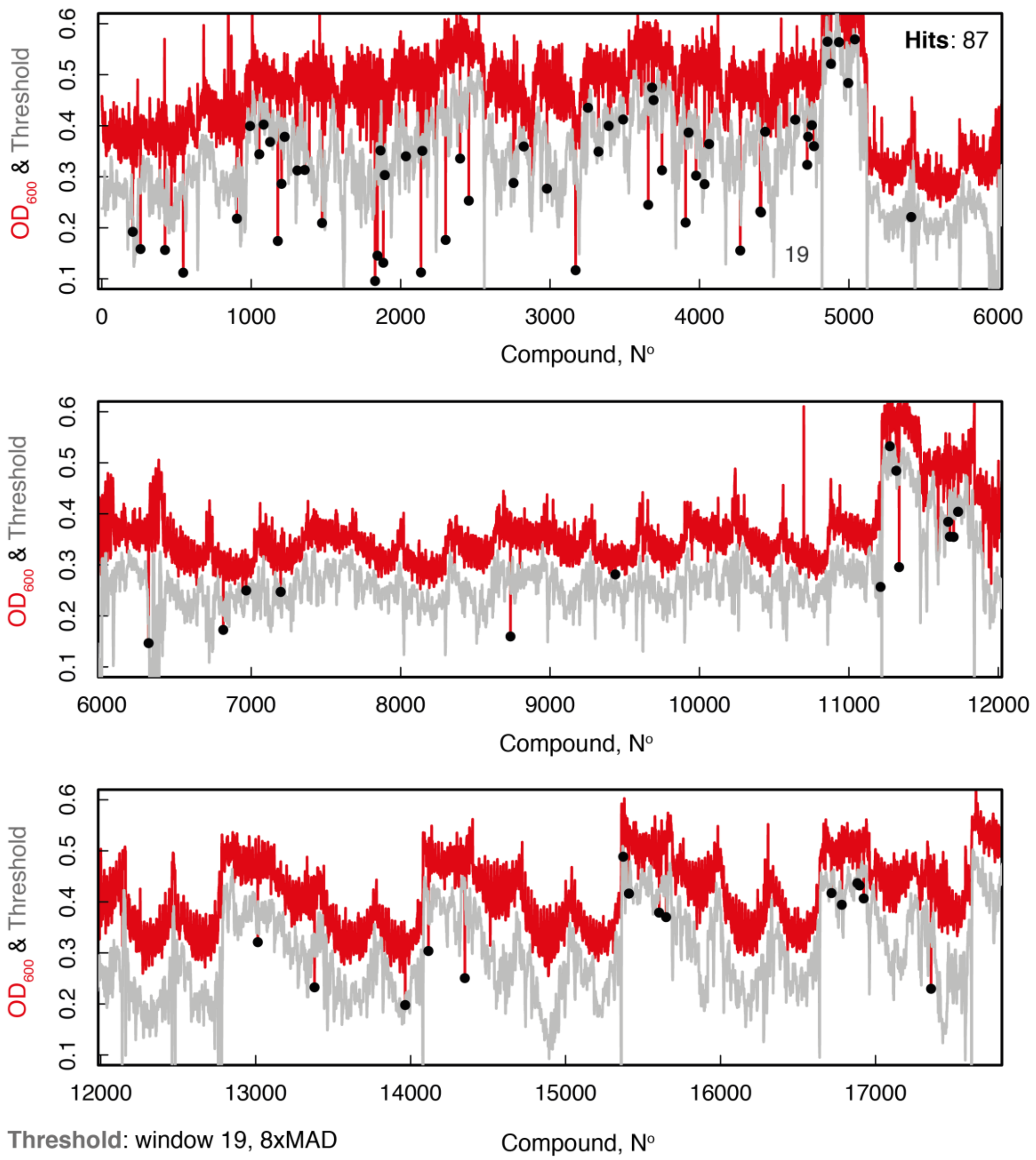

Supplementary Figure 2 | Graphical output of the HTS hit detection script. Red trace represents input (acquired OD_{600} values), grey trace is calculated threshold for every position in a given window. Black filled circles represent OD_{600} value of hits. 87 hits indicated by black dots are acquired with the following settings: window 19, threshold 8xMAD.

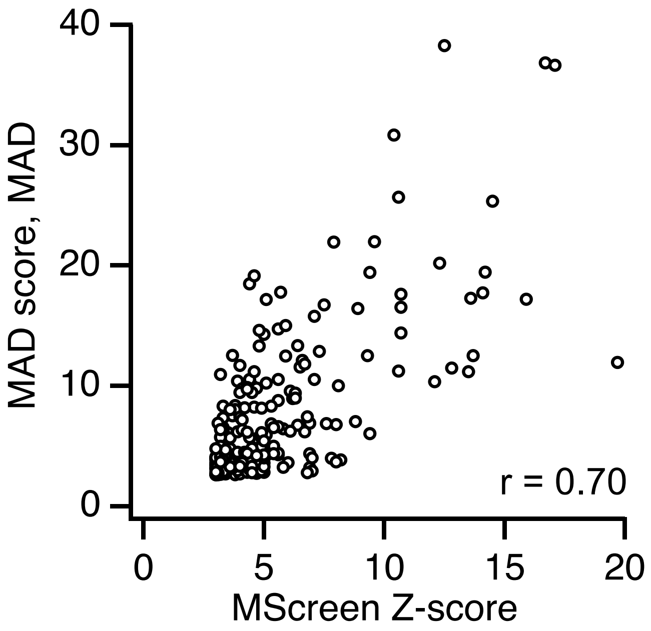

Supplementary Figure 3 | Correlation between MScreen[5] and MAD score strategies used to identify hit compounds in the primary screen. MAD score reflects the OD_{600} deviation of a well from the median OD_{600} calculated for a 19-well sliding window and is expressed in MAD units. Z-score of MScreen[5] reflects the deviation of the OD_{600} of a well from the mean OD_{600} calculated for the whole plate. Both scores take into account only wells with compounds added. r – Pearson's product-moment correlation coefficient.

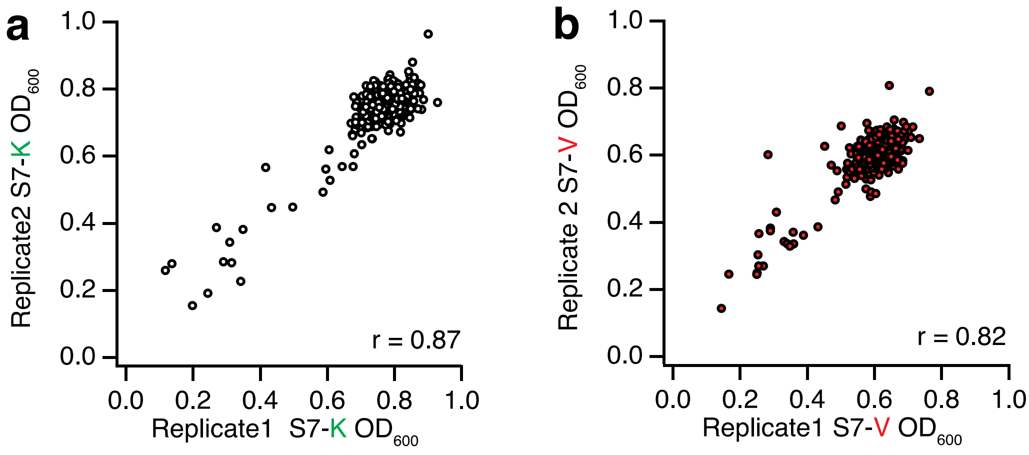

Supplementary Figure 4 | Reproducibility of the two replicates of the secondary screen for Rel inhibitors. 480 hit compounds identified in the primary screen were tested for growth inhibition in S7-K (**a**) and S7-V (**b**) media. ΔSAS *B. subtilis* was grown in S7-V and S7-K liquid media on 384-well plates in the presence of 10 μM test compounds compound and 0.9 % DMSO. OD_{600} values were scored after 9 hours at 37°C. r – Pearson's product-moment correlation coefficient.

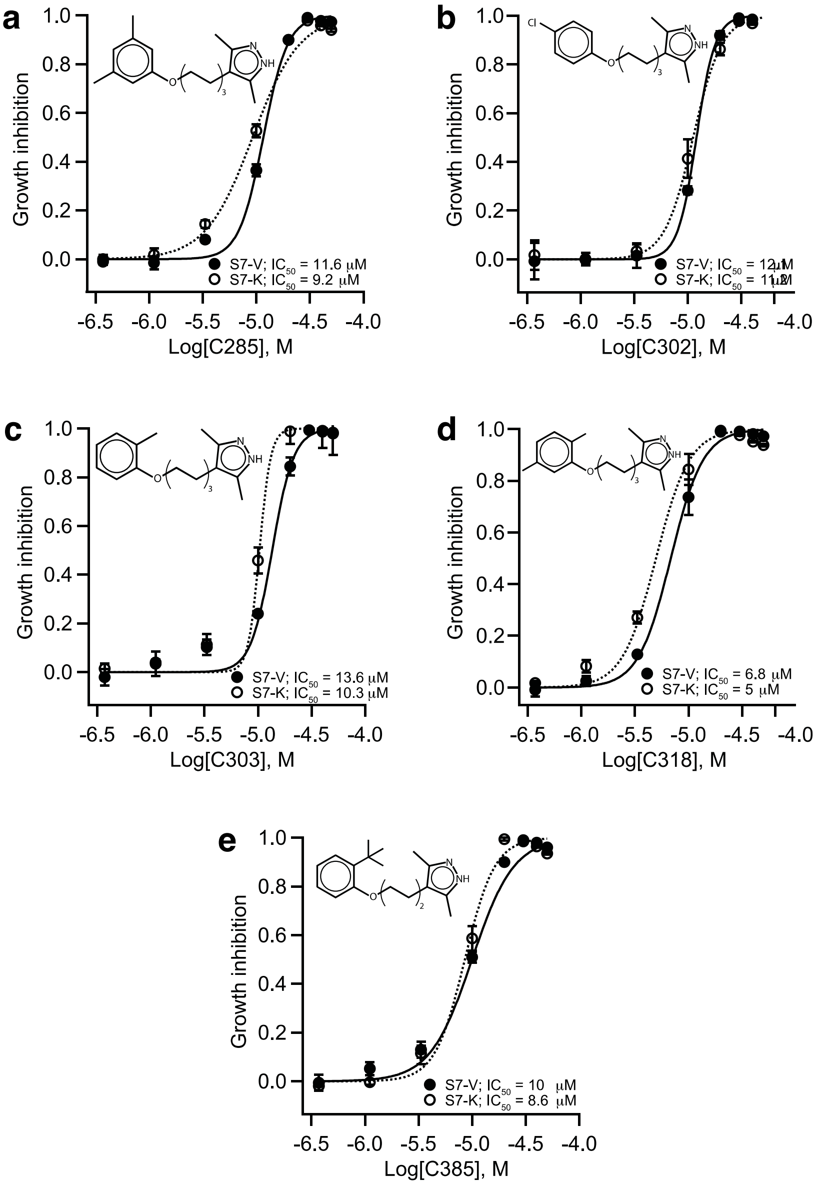

Supplementary Figure 5 | Dose-response curves for the hit compounds. 4-(6-alkyl)-3,5-dimethyl-1H-pyrazoles identified by our HTS as potential stringent response inhibitors were tested in dose-response assay. Compounds were serially diluted in DMSO and tested for growth inhibition in screening conditions, except final concentration of the compound varied from 0 to 50 µM, and the experiments were performed in 96-well format in presence of 1.3% DMSO.

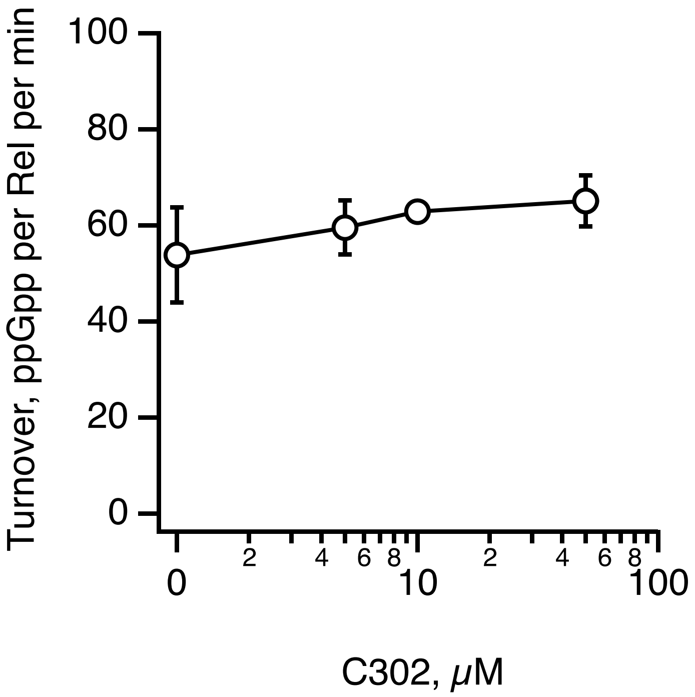

Supplementary Figure 6 | Hit compounds do not inhibit *Bacillus subtilis* Rel ppGpp synthetase activity *in vitro*. ppGpp synthesis reactions in presence of increasing concentrations of C302 (0-50 μM) were performed in 1x HEPES:Polymix buffer (25 mM HEPES, 15 mM Mg^{2+} and 1 mM βME) and contained 0.5 μM *B. subtilis* ribosomes, 100 μM ppGpp, 300 μM ^{3}H-labelled GDP, 0.1 μM Rel, 0.12 μg/ml polyU mRNA, 2 μM $tRNA^{Phe}$. The reaction mixture was pre-incubated at 37°C for 2 min and started by addition of 1 mM ATP. At each time points 5 μl of reaction mixture was quenched with 70% formic acid and analyzed by TLC.

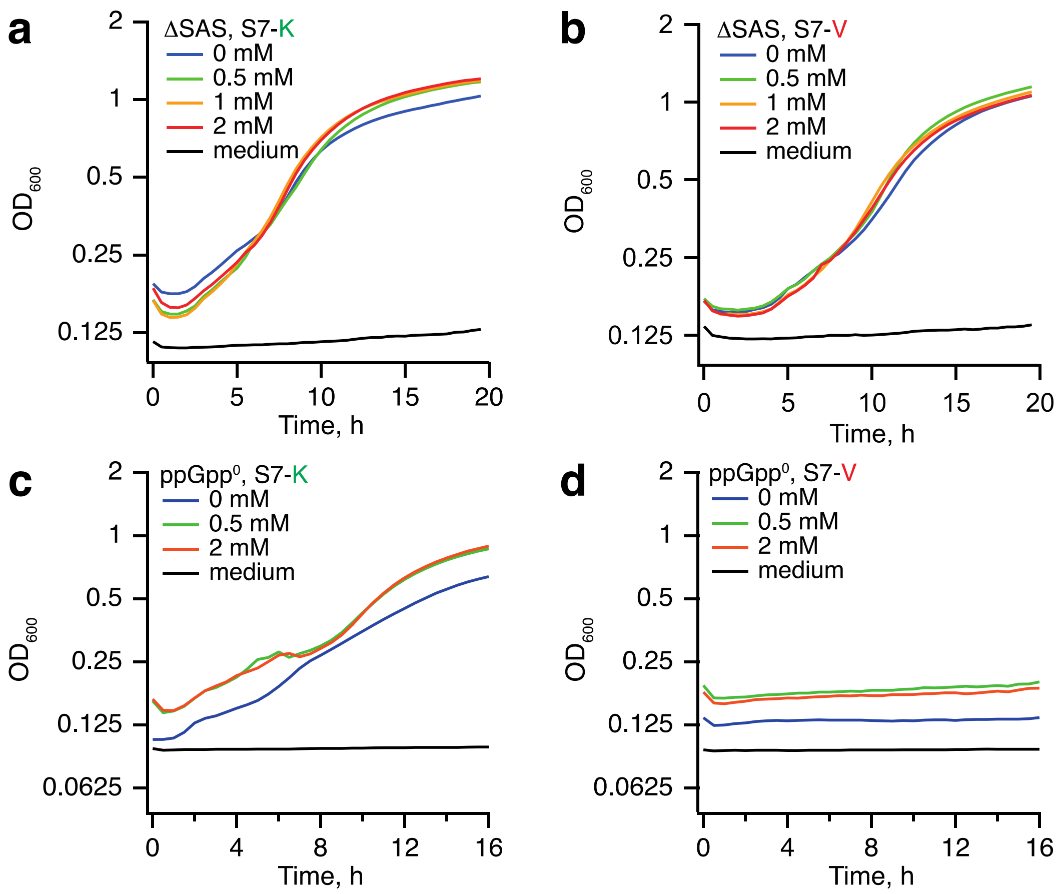

Supplementary Figure 7 | Relacin does not pass the selection criteria for a selective Rel inhibitor of the current auxotrophy-based HTS. Growth of ΔSAS (**a**, **b**) and ppGpp^0 (**b**, **d**) *B. subtilis* was followed in S7-K (**a**, **c**) and S7-V (**b**, **d**) in the presence of increasing concentrations of Relacin[6]. Even at 2 mM Relacin fails to inhibit ΔSAS growth in S7-V (**b**), and has little effect on growth of the ppGpp^0 strain regardless the media (**c**, **d**).

Supplementary References

1. Mechold, U., Murphy, H., Brown, L. & Cashel, M. Intramolecular regulation of the opposing (p)ppGpp catalytic activities of Rel(Seq), the Rel/Spo enzyme from Streptococcus equisimilis. *J Bacteriol* **184**, 2878-88 (2002).
2. Shyp, V. et al. Positive allosteric feedback regulation of the stringent response enzyme RelA by its product. *EMBO Rep* **13**, 835-9 (2012).
3. Nanamiya, H. et al. Identification and functional analysis of novel (p)ppGpp synthetase genes in Bacillus subtilis. *Mol Microbiol* **67**, 291-304 (2008).
4. Zhang, J.H., Chung, T.D. & Oldenburg, K.R. A Simple Statistical Parameter for Use in Evaluation and Validation of High Throughput Screening Assays. *J Biomol Screen* **4**, 67-73 (1999).
5. Jacob, R.T. et al. MScreen: an integrated compound management and high-throughput screening data storage and analysis system. *J Biomol Screen* **17**, 1080-7 (2012).
6. Wexselblatt, E. et al. Relacin, a novel antibacterial agent targeting the Stringent Response. *PLoS Pathog* **8**, e1002925 (2012).
